# Supplementary material for: An excess of niche differences maximizes ecosystem functioning
Source: Nat Commun. 2020 Aug 21;11:4180. doi: 10.1038/s41467-020-17960-5 (PMC7442808; doi:10.1038/s41467-020-17960-5)
Supplement: Supplementary file 1 — Supplementary Information [file 41467_2020_17960_MOESM1_ESM.pdf]

An excess of niche differences maximizes ecosystem functioning

Oscar Godoy, Lorena Gómez-Aparicio, Luis Matías, Ignacio M. Pérez-Ramos, Eric Allan

Supplementary Information

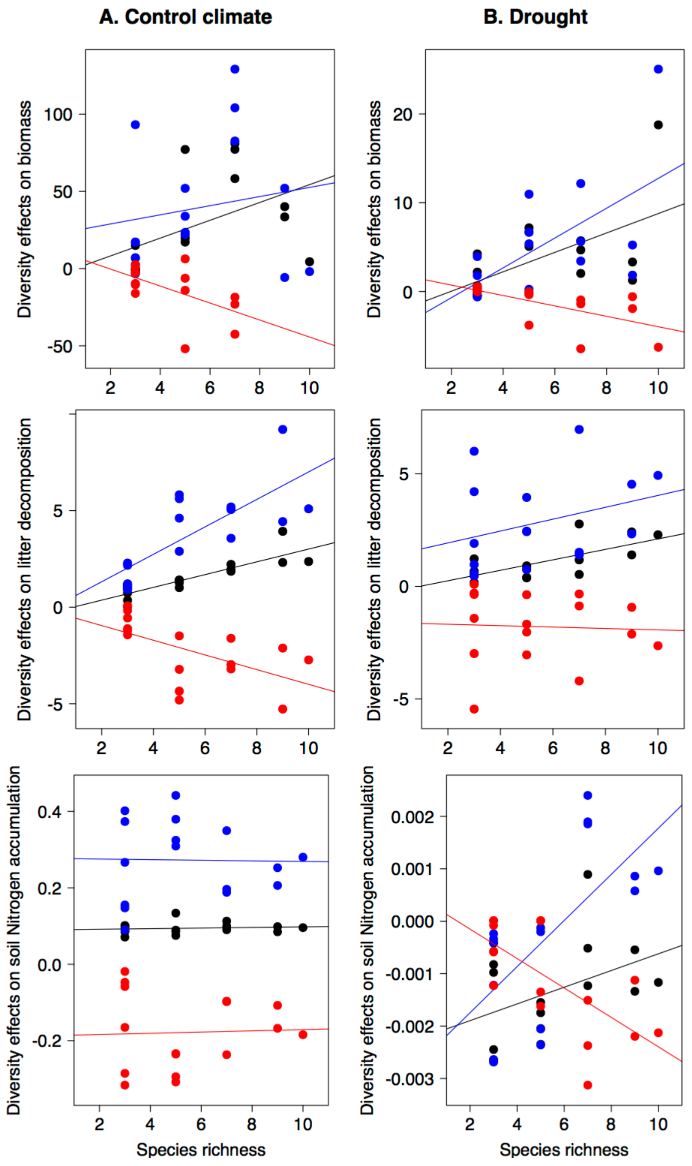

Supplementary Figure 1 Diversity effects on the three functions evaluated. Net biodiversity effects (black line) and its two components (complementarity (blue line) and selection effects (red line)) as a function of species richness across the three functions considered (biomass production, litter decomposition, and soil nutrient accumulation under the two contrasted climatic conditions (A) Control climate and (B) Drought. Species richness was a significant predictor ($p < 0.05$) of the net effect of biodiversity on productivity for all functions except for litter decomposition under drought conditions and soil nitrogen under control climate. For soil nutrients, we represent the particular case of nitrogen but very similar relationships were observed for the other elements considered.

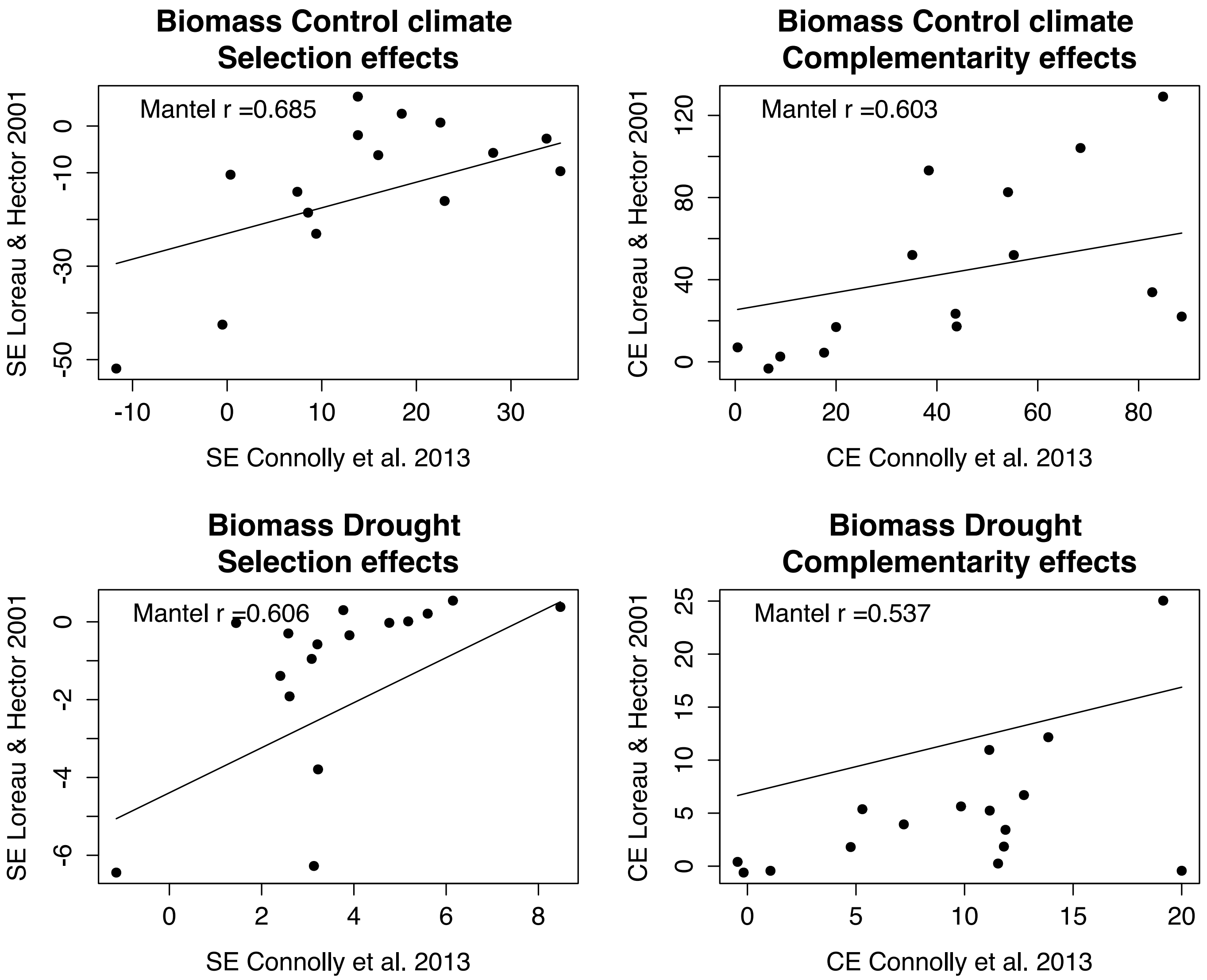
Biomass Control climate
Selection effects
Mantel r =0.685
SE Loreau & Hector 2001
SE Connolly et al. 2013
-50
-30
-10
0
-10
0
10
20
30
Biomass Control climate
Complementarity effects
Mantel r =0.603
CE Loreau & Hector 2001
CE Connolly et al. 2013
0
40
80
120
0
20
40
60
80
Biomass Drought
Selection effects
Mantel r =0.606
SE Loreau & Hector 2001
SE Connolly et al. 2013
-6
-4
-2
0
0
2
4
6
8
Biomass Drought
Complementarity effects
Mantel r =0.537
CE Loreau & Hector 2001
CE Connolly et al. 2013
0
5
10
15
20
25
0
5
10
15
20

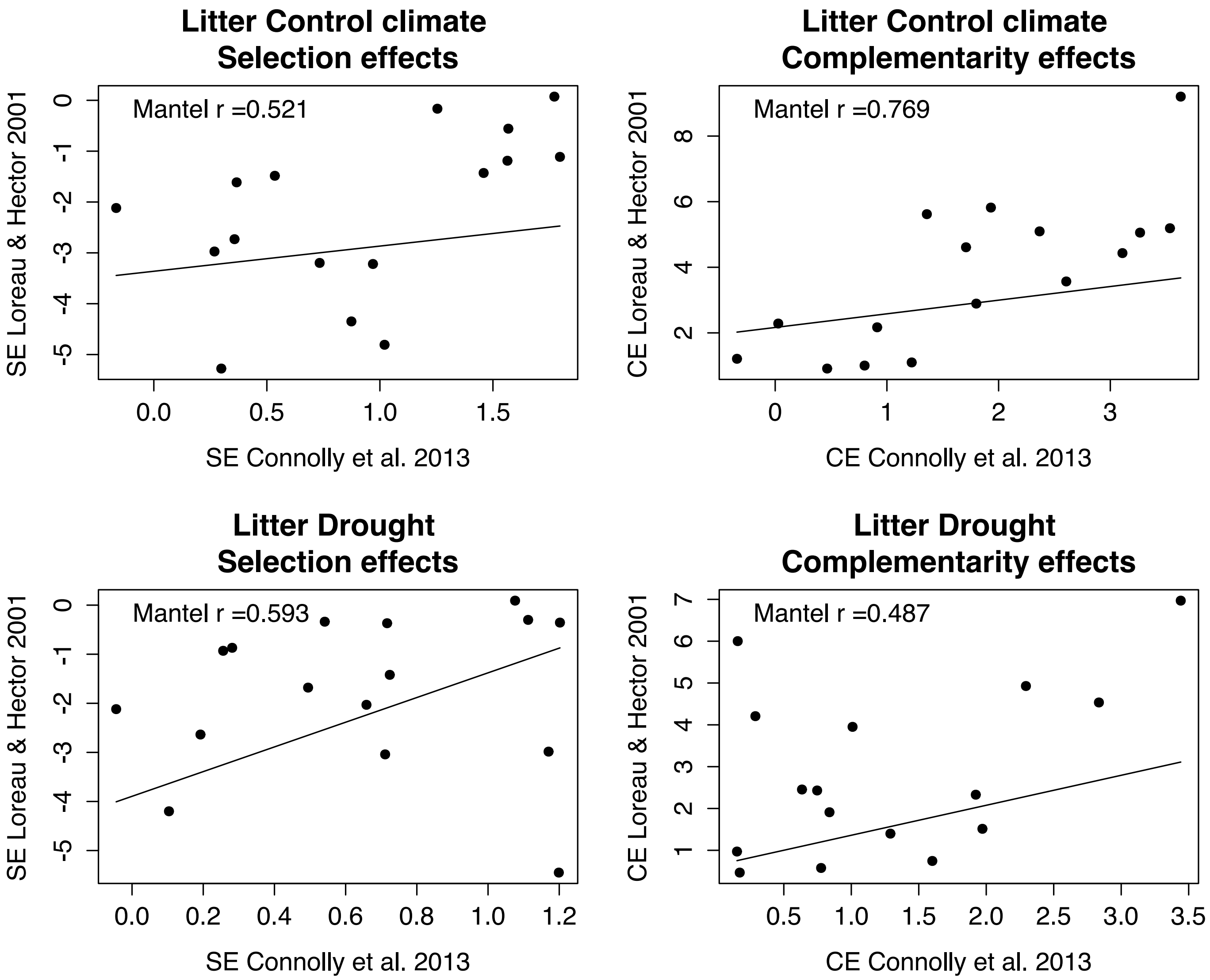

Litter Control climate
Selection effects
Mantel r =0.521
SE Loreau & Hector 2001
SE Connolly et al. 2013
Litter Control climate
Complementarity effects
Mantel r =0.769
CE Loreau & Hector 2001
CE Connolly et al. 2013
Litter Drought
Selection effects
Mantel r =0.593
SE Loreau & Hector 2001
SE Connolly et al. 2013
Litter Drought
Complementarity effects
Mantel r =0.487
CE Loreau & Hector 2001
CE Connolly et al. 2013

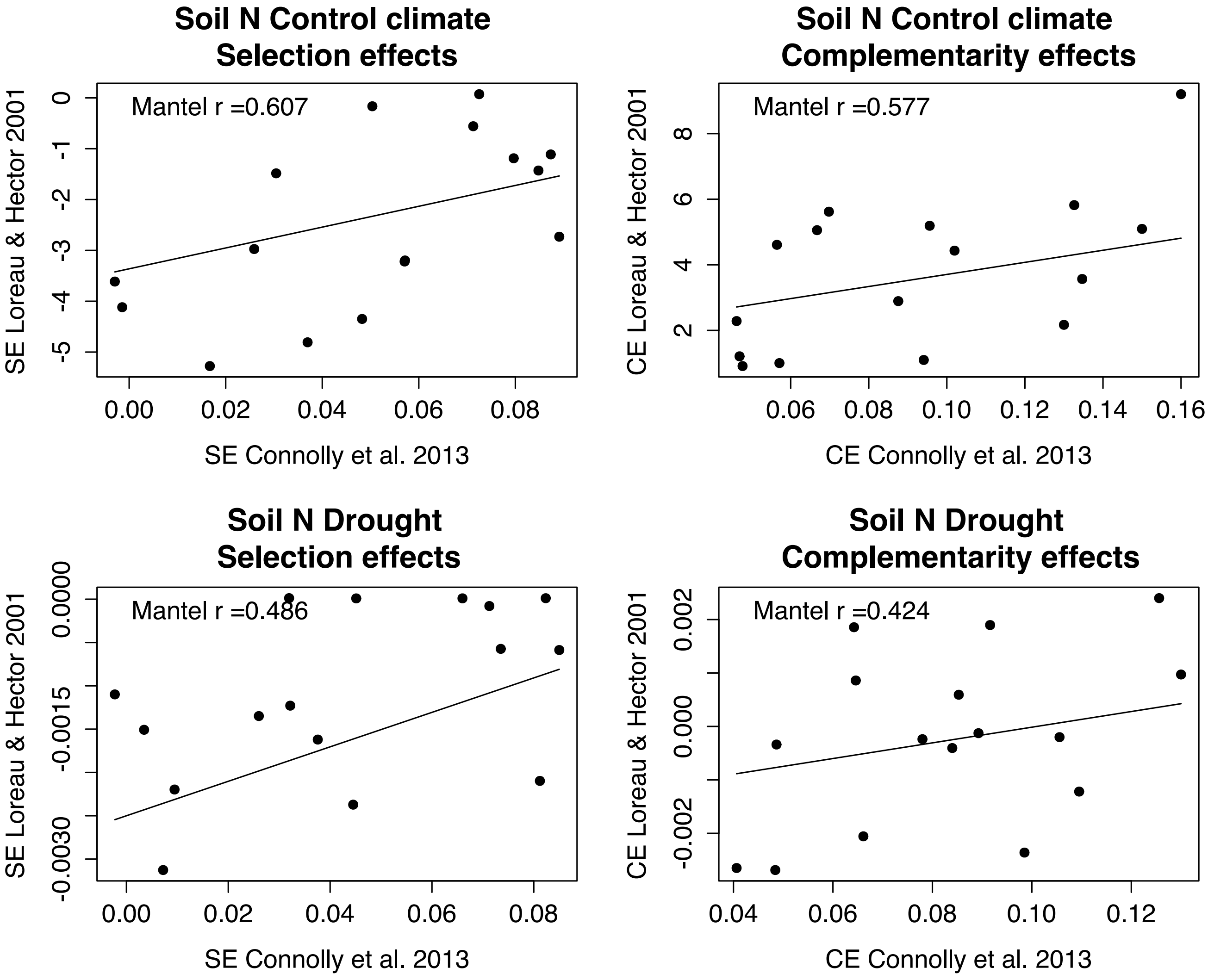

Supplementary Figure 2 Comparison of additive partitioning and diversity interaction models. Correlation between complementarity effects and differences in species' selection effects obtained from additive partitioning (Loreau &Hector 2001) (y-axis) and from diversity interaction models (Connolly et al. 2013) (x-axis). These graphs show that for the multiple functions (biomass, litter decomposition, soil nutrient content) evaluated in our experiment complementarity and selection effects correlated well between the two approaches under control climate (upper panels) and drought conditions (bottom panels). To obtain complementarity and selection effects at the community level using diversity interaction models, we sum all complementarity effects between the species in the community and all selection effects for the individual species.

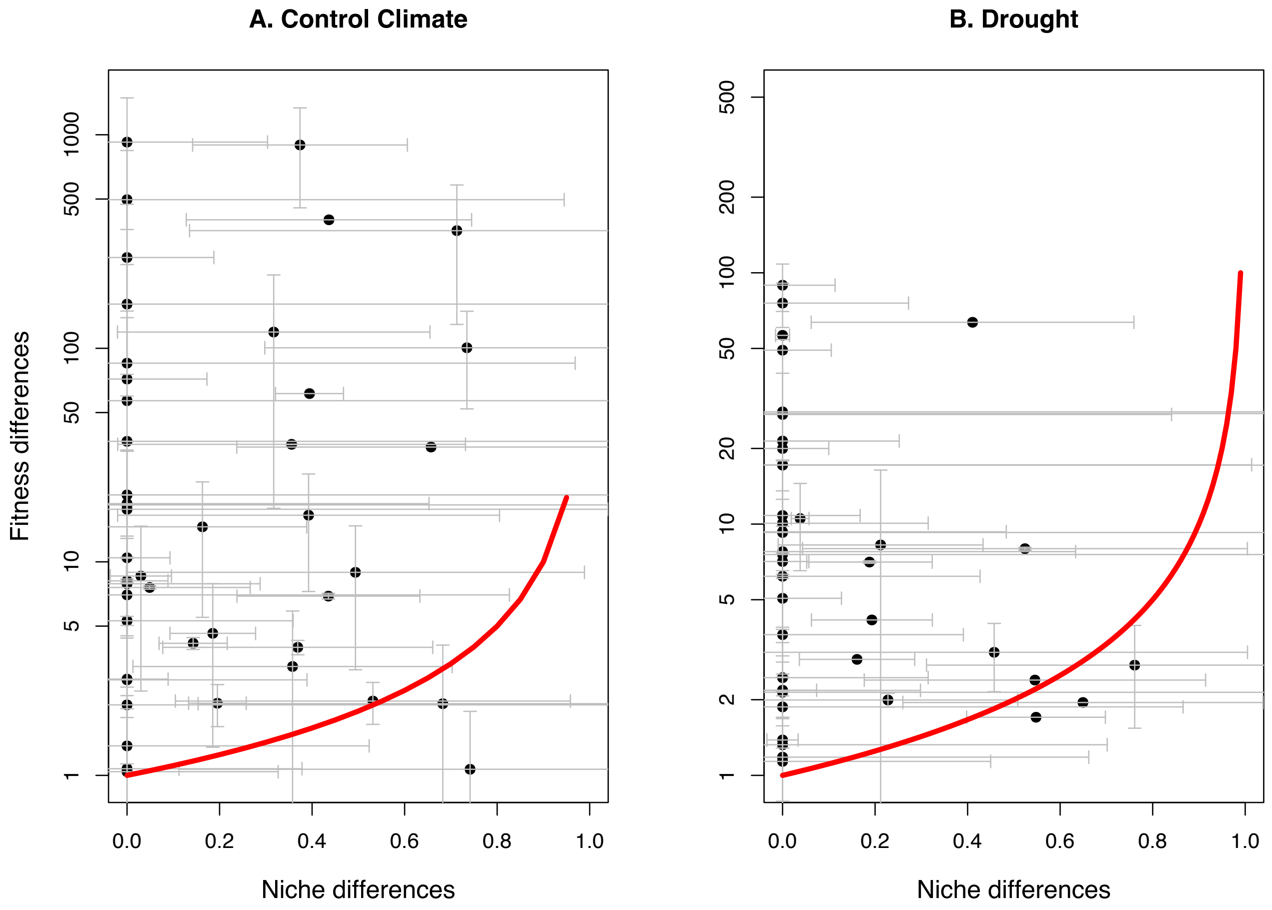

Supplementary Figure 3 Relationship between pairwise niche and fitness differences for the two climate scenarios. (A) control climatic scenario for the study area where the first major rains after the drought period occurred in October, and (B) drought event where first major rains came in December, causing a 2-month delay in seed germination. Note that each point represents a mean of niche and fitness differences for a pair of species, and error bars show its standard deviation. Error bars for each point were obtained from n=16 independent individuals, 8 per species, which correspond to the two independent samples of individual seed production across the four different density treatments. The red solid line separates the region where the condition for coexistence is met ($\rho < \kappa_j/\kappa_i$) from the competitive exclusion region. Three species pairs fall in the coexistence region under control climatic conditions and also three under drought. For the rest of the species pairs, average fitness differences exceed stabilising niche differences. Note that our experiment focused on interactions at the neighborhood spatial scale over a single

generation and therefore does not capture the spatial and temporal heterogeneity that allows these pairs to coexist at the landscape scale.

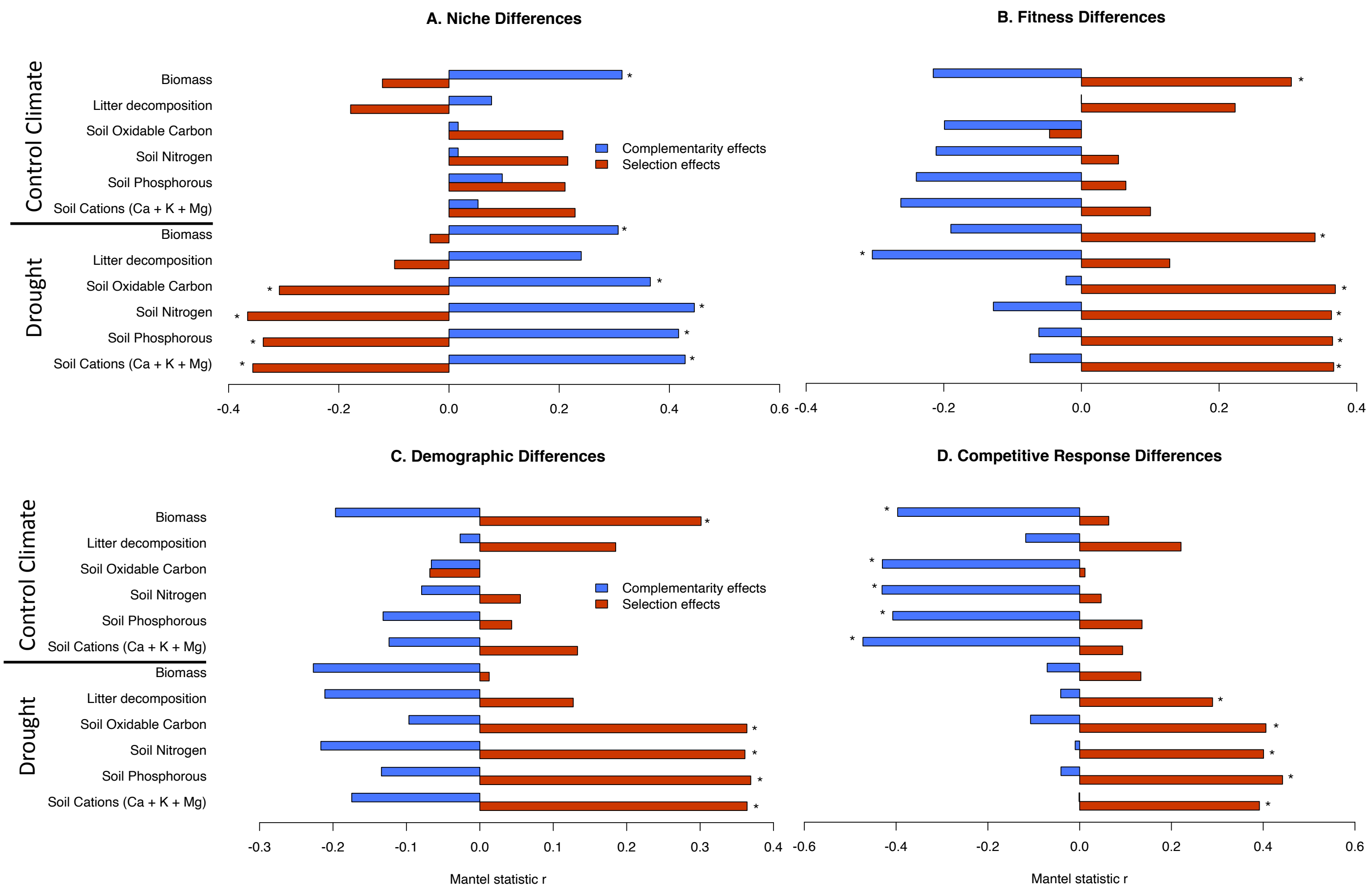

Supplementary Figure 4 Correlations of niche and fitness differences with all functions evaluated. Same as Figure 2 in the main text, this supplementary figure shows correlations between (A) stabilizing niche and (B) average fitness differences and complementarity (blue) and selection effects (red), here for all soil nutrients including soil oxidable C, soil P, and soil cations. Correlations between complementarity and selection are also shown with the two components of fitness differences, the demographic ratio (C) and the competitive response ratio (D). Soil oxidable C, soil P, and soil cations. Correlations are shown for the two climates (control and drought). Significant correlations, following Benjamini-Hochberg correction of raw p-

values for multiple comparisons (n= 24) per species difference evaluated, are marked with an asterisk. Statistical significance involved a two-sided evaluation because it is expected that both complementarity and selection effects can contribute positively and negatively to niche and fitness differences.
