# Supplementary material for: An excess of niche differences maximizes ecosystem functioning
Source: Nat Commun. 2020 Aug 21;11:4180. doi: 10.1038/s41467-020-17960-5 (PMC7442808; doi:10.1038/s41467-020-17960-5)
Supplement: Supplementary file 3 — Reporting Summary [file 41467_2020_17960_MOESM3_ESM.pdf]

nature research

Corresponding author(s): Oscar Godoy

Last updated by author(s): Jun 27, 2020

Reporting Summary

Nature Research wishes to improve the reproducibility of the work that we publish. This form provides structure for consistency and transparency in reporting. For further information on Nature Research policies, see Authors & Referees and the Editorial Policy Checklist .

Statistics

For all statistical analyses, confirm that the following items are present in the figure legend, table legend, main text, or Methods section.

n/a	Confirmed	
☐	☒	The exact sample size (*n*) for each experimental group/condition, given as a discrete number and unit of measurement
☐	☒	A statement on whether measurements were taken from distinct samples or whether the same sample was measured repeatedly
☐	☒	The statistical test(s) used AND whether they are one- or two-sided<br>*Only common tests should be described solely by name; describe more complex techniques in the Methods section.*
☐	☒	A description of all covariates tested
☐	☒	A description of any assumptions or corrections, such as tests of normality and adjustment for multiple comparisons
☐	☒	A full description of the statistical parameters including central tendency (e.g. means) or other basic estimates (e.g. regression coefficient) AND variation (e.g. standard deviation) or associated estimates of uncertainty (e.g. confidence intervals)
☐	☒	For null hypothesis testing, the test statistic (e.g. *F*, *t*, *r*) with confidence intervals, effect sizes, degrees of freedom and *P* value noted<br>*Give P values as exact values whenever suitable.*
☒	☐	For Bayesian analysis, information on the choice of priors and Markov chain Monte Carlo settings
☒	☐	For hierarchical and complex designs, identification of the appropriate level for tests and full reporting of outcomes
☒	☐	Estimates of effect sizes (e.g. Cohen's *d*, Pearson's *r*), indicating how they were calculated

Our web collection on statistics for biologists contains articles on many of the points above.

Software and code

Policy information about availability of computer code

Data collection	Data was collected directly from the field experiment built to understand the links between the determinants of species coexistence and functioning under contrasting environments. No data has been obtained from published sources. Data has been made publicly available at DOI: 10.6084/m9.figshare.12578444
Data analysis	All analyses have been conducted with open software, specifically with R language environment. R Package versions used have been included in the manuscript, and the link to the open source of the custom code used in the study has been provided into the code availability statement.

For manuscripts utilizing custom algorithms or software that are central to the research but not yet described in published literature, software must be made available to editors/reviewers. We strongly encourage code deposition in a community repository (e.g. GitHub). See the Nature Research guidelines for submitting code & software for further information.

Data

Policy information about availability of data

All manuscripts must include a data availability statement. This statement should provide the following information, where applicable:

- Accession codes, unique identifiers, or web links for publicly available datasets
- A list of figures that have associated raw data
- A description of any restrictions on data availability

The data availability statement provides a link to download interactions coefficients, species vital rates and pairwise predicted coexistence. All this information is key to reproduce the main results of the manuscript. It is publicly available at DOI: 10.6084/m9.figshare.12578444

Field-specific reporting

Please select the one below that is the best fit for your research. If you are not sure, read the appropriate sections before making your selection.

☐ Life sciences ☐ Behavioural & social sciences ☒ Ecological, evolutionary & environmental sciences

For a reference copy of the document with all sections, see nature.com/documents/nr-reporting-summary-flat.pdf

Ecological, evolutionary & environmental sciences study design

All studies must disclose on these points even when the disclosure is negative.

Study description	We couple a competition experiment to a biodiversity functioning experiment. This combination was done under two contrasted environmental conditions by delaying when seeds from annual plant species were sowed in the ground. For the competition experiment, we established 180 rectangular plots (0.65 m × 0.5 m) in September 2014 prior to the major autumn rains. We randomly assigned each of 80 plots to be sown with one of the 10 species at a density of 2, 4, 8, or 16 g/m2 of viable seed, giving two replicates per density and per species. Each plot was divided into 20 subplots (a four row by five column array) with a buffer of 2 cm along the edge of the plot. At the center of each subplot, we sowed five viable seeds of one of the 10 species, and germinants were thinned to a single individual per subplot. This design allowed us to measure viable seed production on two focal individuals per species and plot, when competing with different number of neighbors of the same species and each of the other 9 species. We additionally established 10 plots that had the same array but did not include any density treatment in order to measure viable seed production of focal individuals of the 10 species in the absence of competition. For the biodiversity functioning experiment, we established 104 circular plots (0.75m2) in the same area and at the same times as the competition experiment. We randomly assigned each plot to be a monoculture or a mixture of 3, 5, 7, 9 and 10 species. All plots were sown at a total seed density of 15g/m2, and seed mass was evenly divided between the species in mixtures. To create the mixtures, we randomly assembled 6 different communities of 3 species, 4 communities of 5 species, 3 communities of 7 species, and 2 communities of 9 species. These communities, as well as the 10 monocultures and the one 10 species mixture, were all replicated twice within each climatic condition (i.e. climate control and drought).
Research sample	We used ten common annual plants, which naturally co-occur at the study site, for the experiment. These species cover a wide phylogenetic and functional range and include members of six of the most abundant families in the Mediterranean grasslands of southern Spain. Seeds were provided by a local supplier (Semillas silvestres S.L.) from populations located near to our study site. Our experiments were located within an 800 m2 area, which had been previously cleared of all vegetation and which was fenced to prevent mammal herbivory. Landscape fabric was placed between plots to prevent growth of weeds.
Sampling strategy	No sample size calculation was performed. We knew from previous field work experience that a total of 20 species (in our case 10 species under two different treatments), is the maximum number that two people can feasible measure the species species vital rates and interaction coefficients needed to parameterize models of population dynamics. In this case, the challenge of field sampling was even stronger because that was only one part of the study. The other part was to perform a biodiversity functioning experiment, which involve measuring several ecosystem functioning properties (biomass production, litter decomposition, and soil nutrient content across a diversity gradient of 3, 5, 7, 9 and 10 species.
Data collection	Data collection was mainly done by Ignacio M. Perez Ramos, Luis Matías and Oscar Godoy. Data was hand collected following the procedure specified at the study description. All data collected were included into csv. files and stored in cloud services of one of the research institutions (University of Cádiz).
Timing and spatial scale	We set up the experiment in September 2014 where first samples were collected. Specifically, these were soil sample to measure nutrient availability before the experiment. This procedure allows evaluating whether the specific diversity treatments modified soil nitrogen availability at the end of the experiment. After sowing the seed at the end of October, next rounds of samples were collected following species natural growing phenology. For instance, we began collecting leaf litter when individuals start to show first symptoms of senescence which occurred in early spring 2015 (January-February). From early spring to May 2015, we collected information of species vital rates and functioning such as biomass production. Latest measurements of viable seed production, soil seed bank, and soil nutrient availability after plant growth were obtained when all individuals finished their life cycles. This occurred in June-July 2015. In sum, the experiment followed the natural life cycle of these annual plants.
Data exclusions	No data was excluded
Reproducibility	In order to improve transparency and to make the results reproducible, code and data has been made publicly available. Corresponding DOIs has been provided into the code and data statements.
Randomization	We randomly selected the species assemblages from 3, 5, 7, to 9 species in order to design the biodiversity functioning experiment. This was done with a simple function in R called sample () in which each species was considered as a number from 1 to 10. The number of random samples taken obey to the trade-off between obtain different experimental communities and to limit the number of communities to be able to conduct an experiment. For instance, with 5 species the potential number of communities is 252, which is an unfeasible number to experimentally manage.
Blinding	Blinding was not relevant for our study as we needed to identity of species to compute the determinants of species coexistence (niche and fitness differences) and the effects of biodiversity on functioning (complementarity and selection effects).

Did the study involve field work? ☒ Yes ☐ No

Field work, collection and transport

Field conditions	The climate is Mediterranean, with mild, wet winters and hot, dry summers. Soils are loamy with pH = 7.74, C/N = 8.70 and organic matter = 1.16% (0-10 cm depth). Precipitation totaled 532 mm during the experiment (September 2014-August 2015), similar to the 50-y average.
Location	Our experiment was conducted at the La Hampa field station of the Spanish National Research Council (CSIC) in Seville, Spain (37°16'58.8"N, 6°03'58.4"W), 72 m above sea level.
Access and import/export	As I have said above, the experiment was conducted in a field station (La Hampa) that belongs to IRNAS-CSIC and is located in the village of Coria del Rio (Spain). We have the approval from the director that we could run our experiments there. The field station mostly do research of olive tree growth and olive production but is has unused areas in which naturally occur Mediterranean annual plant prairies. We planted in one of the areas seeds of species that are commonly found in the field extension, so we did not bring new species to the field stations following the rules of use of IRNAS-CSIC. Samples collected were related to seed production, biomass production, litter on litter bags and soil samples to analyze several nutrients. All samples were tagged with an unique ID and brought to the lab, which is located in Seville city 20km far away from the field station. No specific methods were used for transporting the samples as they do not involve any risk for human health.
Disturbance	We did not create any major disturbance to the field site besides clearing weeds by removing them manually before sowing in the ground the mixtures of seeds needed to run the experiment.

Reporting for specific materials, systems and methods

We require information from authors about some types of materials, experimental systems and methods used in many studies. Here, indicate whether each material, system or method listed is relevant to your study. If you are not sure if a list item applies to your research, read the appropriate section before selecting a response.

Materials & experimental systems

n/a	Involved in the study
☒	☐ Antibodies
☒	☐ Eukaryotic cell lines
☒	☐ Palaeontology
☒	☐ Animals and other organisms
☒	☐ Human research participants
☒	☐ Clinical data

Methods

n/a	Involved in the study
☒	☐ ChIP-seq
☒	☐ Flow cytometry
☒	☐ MRI-based neuroimaging
